# Evidence for transovarial transmission of tick-borne rickettsiae circulating in Northern Mongolia

**DOI:** 10.1371/journal.pntd.0006696

**Published:** 2018-08-27

**Authors:** Thomas C. Moore, Laura A. Pulscher, Luke Caddell, Michael E. von Fricken, Benjamin D. Anderson, Battsetseg Gonchigoo, Gregory C. Gray

**Affiliations:** 1 Division of Infectious Diseases and Duke Global Health Institute, Duke University, Durham, North Carolina, United States of America; 2 Miller School of Medicine, University of Miami, Miami, Florida, United States of America; 3 Department of Global and Community Health, George Mason University, Fairfax, Virginia, United States of America; 4 Institute of Veterinary Medicine, Ulaanbaatar, Mongolia; Baylor College of Medicine, UNITED STATES

## Abstract

Transstadial transmission of tick-borne rickettsiae has been well documented. Few studies, however, have evaluated the role of transovarial transmission of tick-borne rickettsiae, particularly in nature within the host-vector ecosystem. This cross-sectional study aimed to understand the role of transovarial transmission of tick-borne rickettsiae among feeding ticks at different life stages. Tick eggs laid by engorged wild-caught adult female ticks were pooled and tested for *Rickettsia* spp. and *Anaplasma*/*Ehrlichia* spp. using molecular techniques, while adult fed ticks were tested individually. Additionally, larval and nymphal ticks were collected in the wild from small mammals, pooled and tested for *Rickettsia* spp. and *Anaplasma*/*Ehrlichia* spp. There were 38 fed adult and 618 larvae/nymphs (60 pools total) *Dermacentor* spp. ticks collected from livestock and rodents. All individual adult ticks and tick pools were positive for *Rickettsia* spp. While none of the larvae/nymphs were positive for *Anaplasma*/*Ehrlichia* spp., two adult fed ticks were positive. *Rickettsia* spp. DNA was detected in 91% (30/33) of the pooled eggs tested, and one pool of eggs tested positive for *Anaplasma/Ehrlichia* spp. Sequencing data revealed *Rickettsia* spp. shared ≥99% identity with *R*. *raoultii ompA*. *Anaplasma/Ehrlichia* spp. shared ≥89% identity with *A*. *ovis* 16S ribosomal RNA. This study identified potential transovarial transmission of *Rickettsia* spp. and *Anaplasma* spp. among *D*. *nuttalli* ticks. Additional studies are needed to further assess the proportion of transovarial transmission occurring in nature to better understand the burden and disease ecology of tick-borne rickettsiae in Mongolia.

## Author summary

In this study, we evaluate the probability or likelihood that tick-borne rickettsiae might be transmitted vertically from wild engorged adult female ticks collected throughout the Northern region of Mongolia during the summer of 2016. While significant effort has been directed to study tick-borne rickettsiae, this public health challenge is complicated by the limited knowledge and understanding of tick and tick-borne rickettsiae ecology within Mongolia. Tick-borne rickettsiae of concern to humans and animals in this region of the world are *Rickettsia* spp., *Anaplasma* spp., and *Ehrlichia* spp. Using molecular techniques, we detected rickettsiae among all *Dermacentor* spp. tick life stages and demonstrated potential vertical transmission of *Rickettsia* spp., and *Anaplasma* spp. among wild engorged adult female *Dermacentor nuttalli* ticks. We believe our findings provide important information regarding the ecology of key rickettsiae associated with tick-borne disease in Mongolia.

## Introduction

While significant effort has been directed to study tick-borne rickettsiae, they continue to be a global public health threat. Mongolia is a country known for its rich nomadic and pastoral culture, with populations of people who work very closely with their livestock in environments that are often densely populated with ticks. Additionally, ecotourism is a rapidly growing industry in Mongolia, placing international visitors at risk of exposure to tick-borne rickettsiae [1, 2]. This public health challenge is further complicated by a limited knowledge and understanding of tick and tick-borne rickettsiae ecology within Mongolia [2–4].

The mobility of ticks is restricted to questing and travelling via feeding on animals and humans [5]. Tick-borne rickettsiae typically undergo transstadial transmission before being vectored by a competent tick host. However, depending on the tick species and the type of tick-borne rickettsiae, transovarial transmission may also occur [6]. Research related to transovarial transmission has been particularly limited within the Asian and Eurasian regions of the world.

Several tick-borne rickettsiae surveillance and case studies have been conducted throughout China, Russia and Mongolia, which have tested humans [7–10], livestock [11–15], wildlife [16–19], and ticks [20–28]. However, most of these studies focused exclusively on ticks in their adult life stage, either fed or unfed. Few studies have examined the larval and nymphal stages of ticks in the Eurasian environment. Larval and nymphal life stages of ticks are of special interest in regard to exposure risk, as their small size can lead to less readily detectable feeding on human hosts [29–31].

Tick-borne rickettsiae of most concern in the Asian and Eurasian regions of the world are *Rickettsia* spp. [21, 24], *Anaplasma* spp. [12, 14, 16, 25, 27, 32], and *Ehrlichia* spp. [17, 25]. These rickettsiae have been associated with small mammal reservoirs [6, 17, 19]. Collectively, the objectives of this study were to further investigate the life cycle of tick-borne rickettsiae in locally occurring ticks; to examine the propensity of certain tick-borne rickettsiae to undergo potential transovarial transmission; and to evaluate the infection prevalence of tick-borne rickettsiae infections from early life stage ticks throughout the Northern Mongolia region.

## Methods

**Fig 1:**
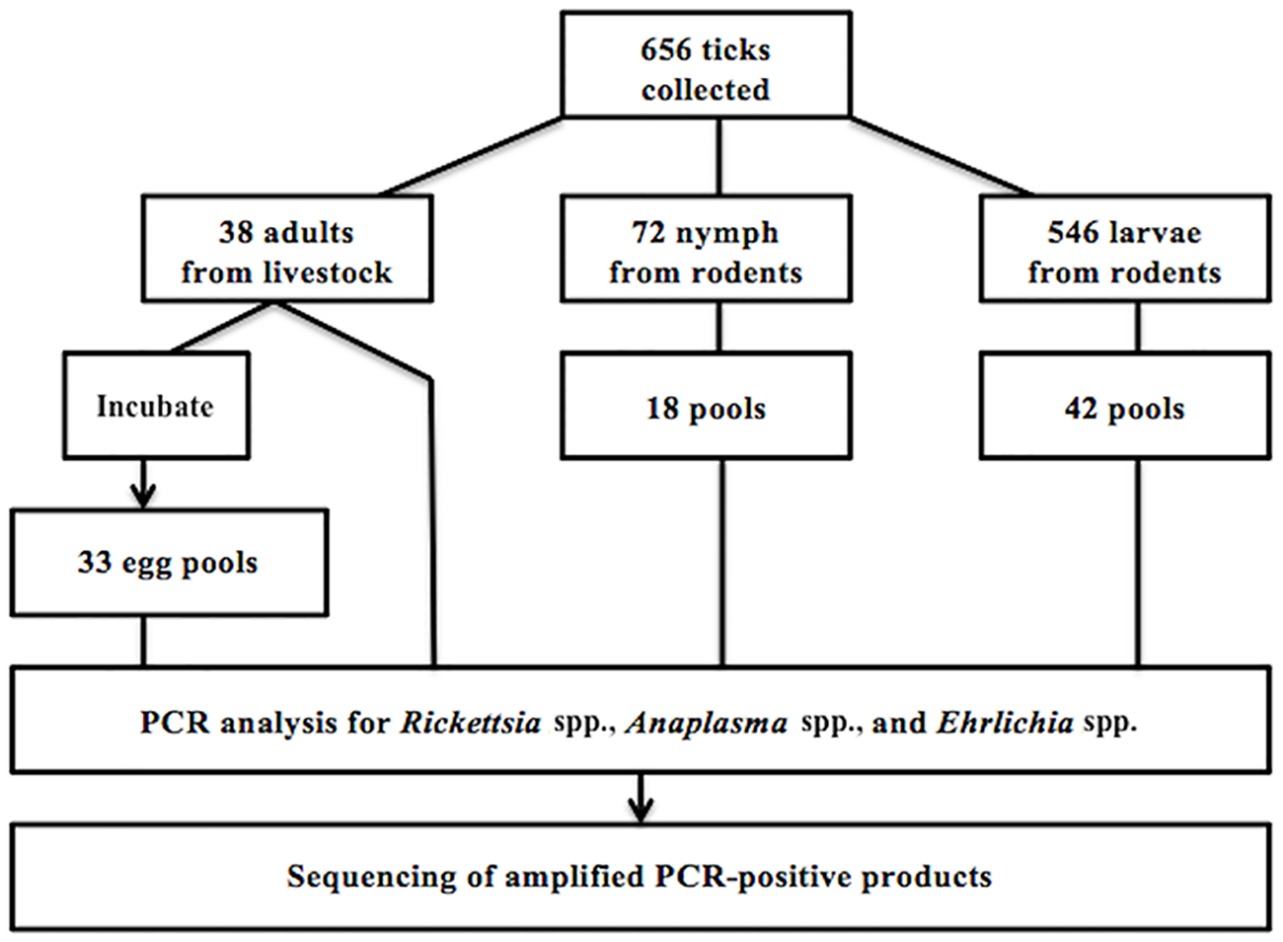
Summary of study design.

**Fig 2:**
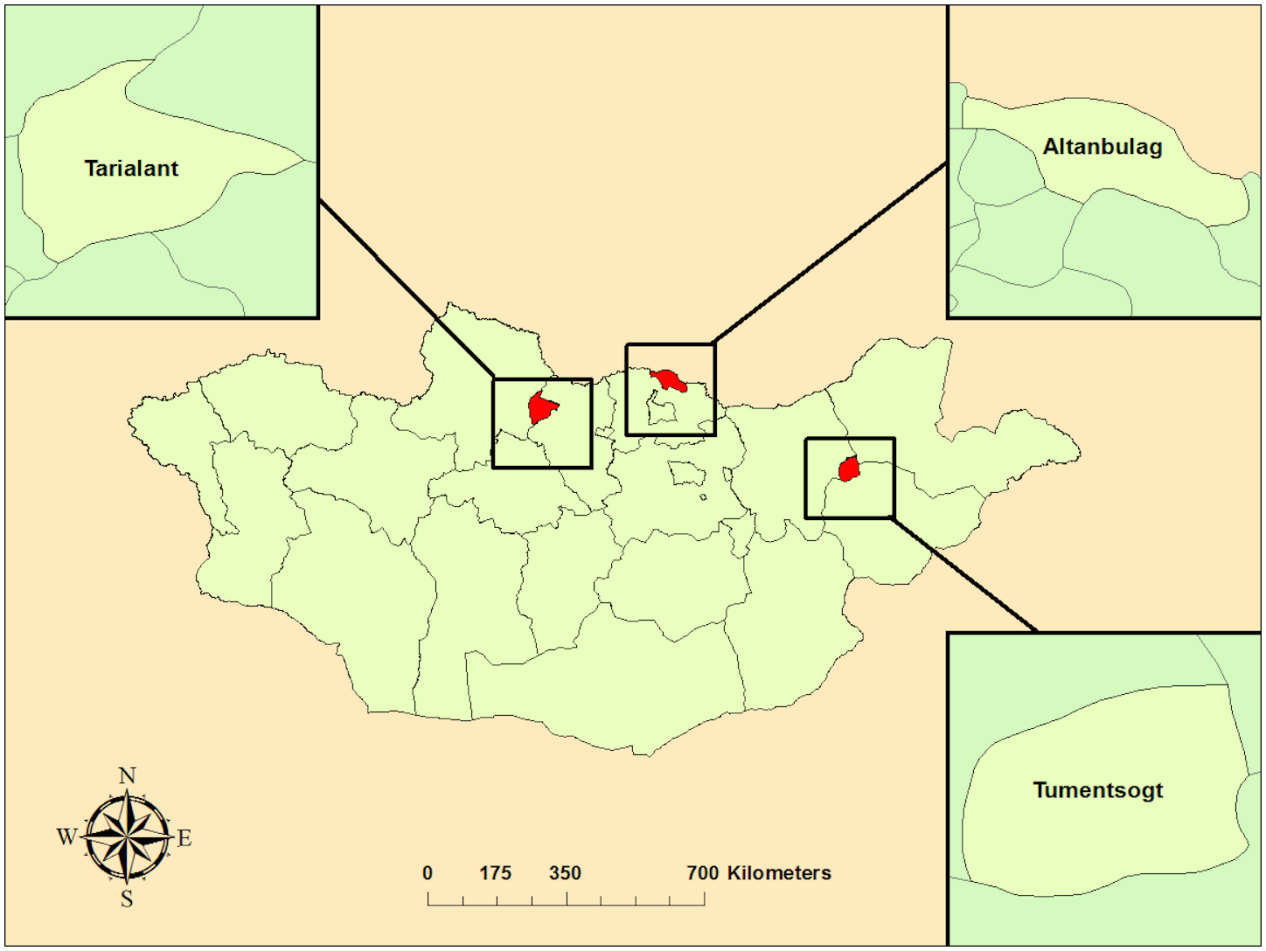
Map of soums where engorged ticks were collected in Mongolia. Soums of tick collection sites were highlighted using ArcGIS 10.4 (ESRI, Redlands, CA). Maps were downloaded from Mongolian Environmental Health Geodatabase (http://www.eic.mn/).

**Fig 3:**
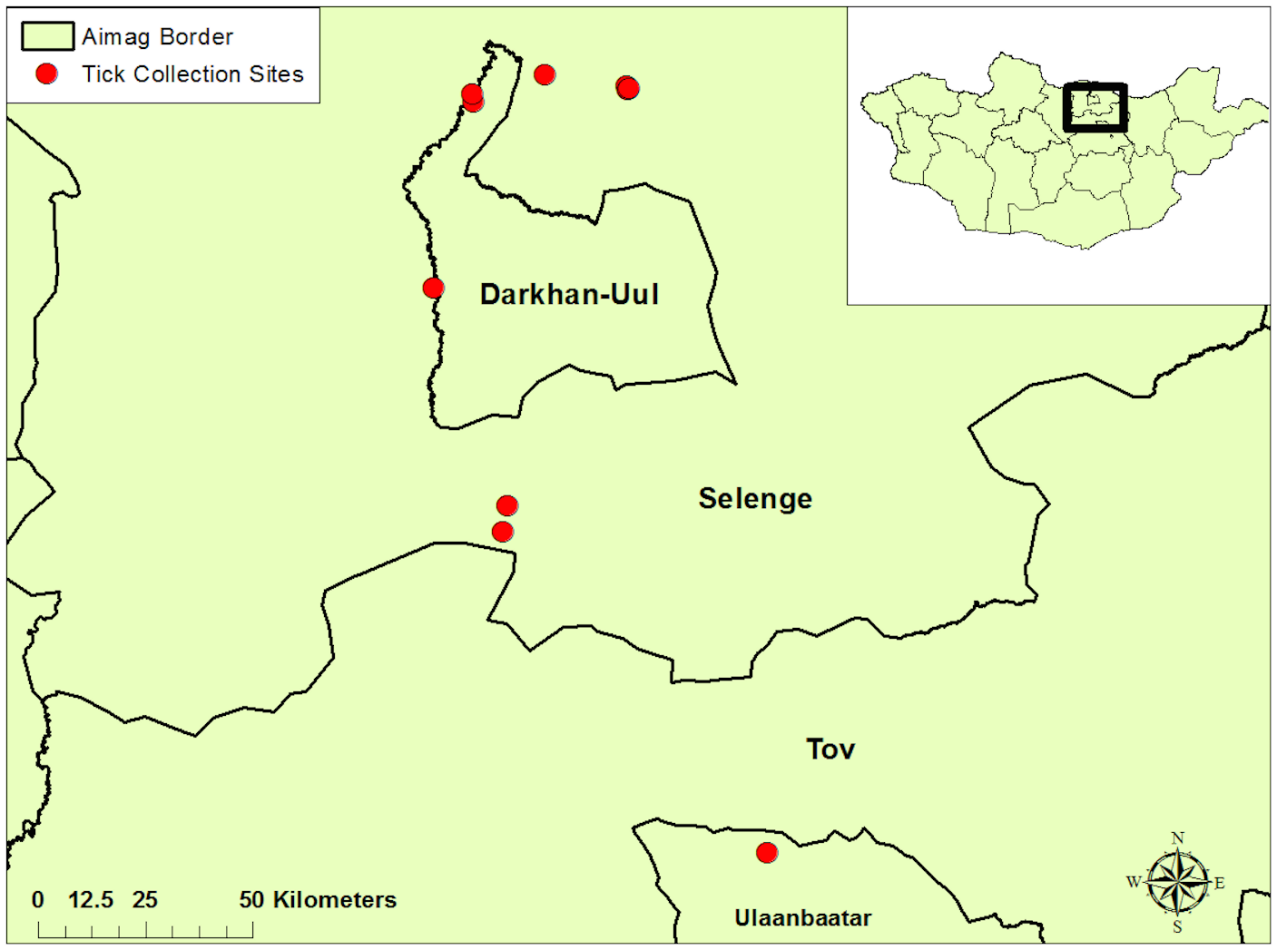
Map of larval/nymphal tick collection sites in Mongolia. GPS data points of tick collection sites were downloaded into ArcGIS 10.4 (ESRI, Redlands, CA). Maps were downloaded from Mongolian Environmental Health Geodatabase (http://www.eic.mn/).

This cross-sectional study was designed to evaluate ticks at different life stages (Fig 1). First, engorged adult ticks were collected from livestock located in three soums (counties) within three aimags (provinces) (Fig 2) from May 6^th^ to 22^nd^, 2016. The second component of this study, examined larvae and nymphs removed from trapped small mammals across seven soums in three aimags situated in the Northern region of Mongolia from June 20^th^ to July 23^rd^, 2016. Using a handheld global positioning system (GPS) unit (Juno Trimble Positions System, Sunnyvale, CA), latitudinal and longitudinal coordinates were collected at each small mammal collection site (Fig 3).

### Adult tick and egg collection

Handling procedures for livestock were conducted by trained veterinary staff prior to this study during animal care and were in accordance with the Mongolian Institute of Veterinary Medicine, Ulaanbaatar, Mongolia. Verbal consent was obtained from livestock owners at time of tick collection. Female adult fed ticks were collected from livestock at time of veterinary care of livestock and kept alive at room temperature in storage containers at the Laboratory of Arachno-Entomology and Protozoolgy, Institute of Veterinary Medicine in Ulaanbaatar, Mongolia. Moist cotton was placed near the ventilation of the containers to replicate environmental humidity conditions. Once female ticks laid eggs (between 2–7 days of incubation), both adult ticks and eggs were stored separately at -80°C until DNA extraction was performed. The whole egg clutch was pooled and tested from each adult female tick. Mass of egg clutches ranged from 10 to 410 milligrams. Eggs and adult ticks were briefly rinsed with 70% ethanol in sterile 1 mL vials to remove contamination and then air dried on a sterile dish in preparation for processing [33, 34].

### Larval/nymphal tick collection

Trapping and handling procedures for small mammals were approved by the Duke University Institutional Animal Care and Use Committee (#A086-16-04) in accordance with the Mongolian Institute of Veterinary Medicine, Ulaanbaatar, Mongolia. At each location, live Tomahawk and Sherman traps were placed near holes where there were signs of recent small mammal habitation. All captured small mammals were sedated with ketamine (50 mg/Kg) and inspected for ticks. Ticks were stored in 70% ethanol at room temperature. Specimens were taxonomically identified to genus for larvae and nymphs and species for adults by a trained entomologist. Ticks were air dried and pooled based on life stage (larvae range 1–15; nymphs range 1–5), small mammal host, sampling location, and tick genus. Pools (n = 60) were stored at -20°C in new sterile 1 mL vials before genomic DNA was extracted.

### Polymerase chain reaction and sequencing

All ticks and eggs were ground using a sterile pre-chilled mortar and pestle with 500 μL of sterile PBS and 50 mg sterile sand for friction [35]. Contents were then centrifuged in a 1.5 mL vial at 9,500 g for 5 minutes. Supernatant was pipetted from the sand deposit, inserted into a new vial and stored at -20°C. Genomic DNA was extracted from tick supernatant using TIANamp Genomic DNA Kit (Tiangen Biotech (Beijing) Co., LTD, Beijing, China) and tested for molecular detection of *Rickettsia* spp. targeting the citrate synthase gene (*gltA*) [36] and the outer-membrane protein gene (*ompA*) [37], as previously described (Table 1). For the molecular detection of *Anaplasma* spp. and *Ehrlichia* spp., the 16S rRNA gene [17] was targeted as previously described (Table 1).

**Table 1:** Summary of primers used in molecular assays.

Rickettsiae	Gene	Primers	Sequence (5'—3')	Amplicon Size	Ref.
*Rickettsia* spp.	*gltA*	CS2d	ATG ACC AAT GAA AAT AAT AAT	381bp	[36]
		CSEndr	CTT ATA CTC TCT ATG TAC A		
		RpCS877p	GGG GAC CTG CTC ACG GCG G		
		RpCS1258n	ATT GCA AAA AGT ACA GTG AAC A		
	*ompA*	Rr190.70p	ATG GCG AAT ATT TCT CCA AAA	346bp	[37]
		Rr190.602n	AGT GCA GCA TTC GCT CCC CCT		
		190.70-38s1	AAA ACC GCT TTA TTC ACC		
		190.602-384r1	GGC AAC AAG TTA CCT CCT		
*Anaplasma/ Ehrlichia* spp.	16S rRNA	Ehr1	AAC GAA CGC TGG CGG CAA GC	524bp	[17]
		Ehr2	AGT AYC GRA CCA GAT AGC CGC		
		Ehr3	TGC ATA GGA ATC TAC CTA GTA G		
		Ehr4	CTA GGA ATT CCG CTA TCC TCT		

Gel electrophoresis was used to evaluate amplified products using 1% (w/v) agarose gels stained with ethidium bromide at 120 V. Gels were analyzed using the Gel Doc EZ System (Bio-Rad, Hercules, California) with ultra-violet illumination.

A representative subset of positive amplicons were selected and directly sequenced using Sanger sequencing (Eton Biosciences, Inc., NC, USA). Sequencing results were then compared against the NCBI nucleotide database using the Standard Nucleotide BLAST application (http://www.ncbi.nlm.nih.gov/BLAST/) for species identification. *Rickettsia* spp. *gltA* and *ompA* sequences were used as confirmation of amplified *Rickettsia* spp. samples and *Anaplasma/Ehrlichia* spp. 16S rRNA sequences were used as confirmation of amplified *Anaplasma* spp. *Anaplasma/Ehrlichia* spp., and *Rickettsia* spp. sequences were structured for phylogenetic relatedness using Molecular Evolutionary Genetics Analysis (MEGA) software, version 7.0.

### Data analysis

Engorged tick infection status was compared to corresponding oviposited egg infection status for PCR-positive *Rickettsia* spp. and *Anaplasma/Ehrlichia* spp. samples, as well as sequence data. Transovarial transmission was considered to have occurred when the corresponding female tick and egg mass were both PCR positive.

Statistical analyses, including two-way frequencies with measures of association, were conducted using STATA 14.1 (StataCorp, College Station, TX).

## Results

A total of 656 ticks were collected from 15 different locations across nine soums in five aimags. All ticks were morphologically identified as *Dermacentor* spp. Due to the size of larval and nymphal ticks collected, and the variety of *Dermacentor* spp. found in Mongolia, early life stage ticks were only identified to genus. All adult-fed ticks were morphologically identified to be *D*. *nuttalli*. Of the early life stage ticks collected from small mammals, 546 (88%) were larvae, 72 (12%) were nymphs. There were 588 (95%) of 618 ticks that were allocated into 42 larval and 18 nymphal pools (60 pools total). A total of 38 adult fed female ticks were collected from sheep and cattle. Of the 38 adult ticks collected, 33 laid eggs.

### Molecular results

**Fig 4:**
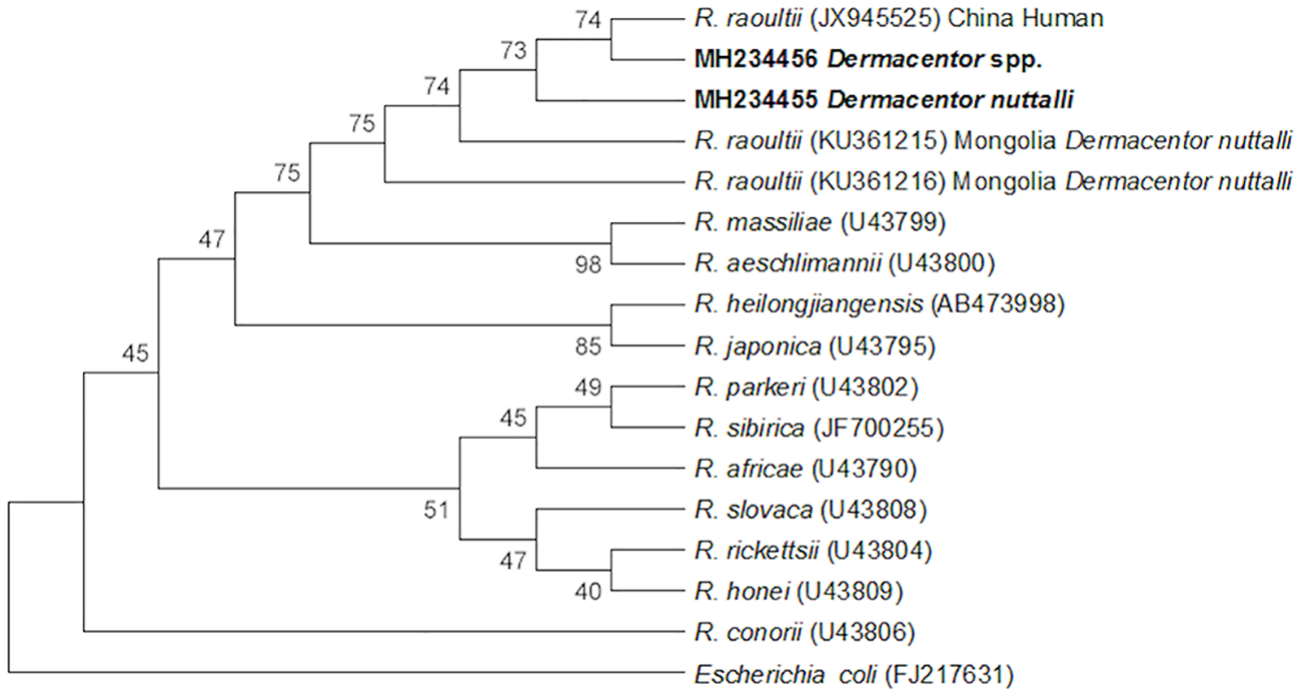
Evolutionary relationships of *Rickettsia* spp. *ompA*. The evolutionary history was inferred using the Neighbor-Joining method [38]. The bootstrap consensus tree inferred from 10,000 replicates is taken to represent the evolutionary history of the taxa analyzed [39]. Branches corresponding to partitions reproduced in less than 50% bootstrap replicates are collapsed. The percentage of replicate trees in which the associated taxa clustered together in the bootstrap test (10,000 replicates) are shown next to the branches [39]. The evolutionary distances were computed using the Kimura 2-parameter method and are in the units of the number of base substitutions per site [40]. The analysis involved 17 nucleotide sequences. Codon positions included were 1st+2nd+3rd+Noncoding. All ambiguous positions were removed for each sequence pair. There were a total of 642 positions in the final dataset. Evolutionary analyses were conducted in MEGA7 [41].

**Fig 5:**
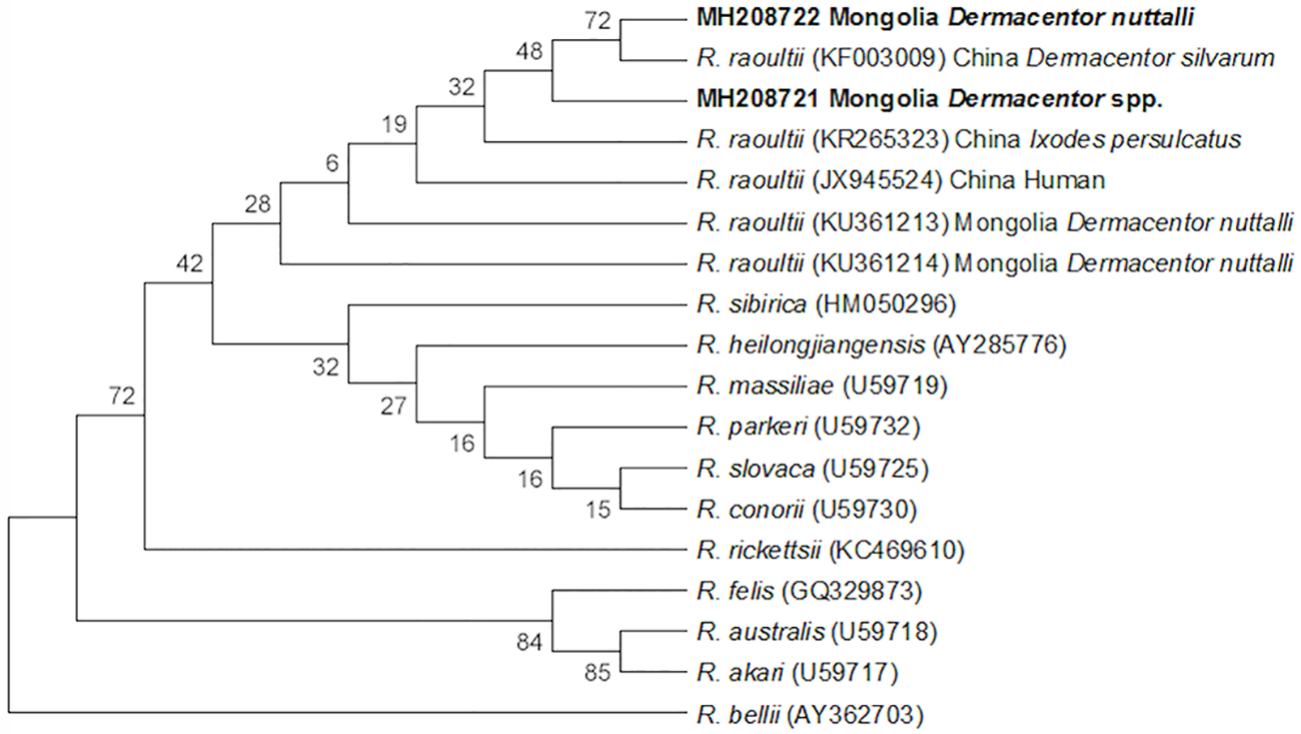
Evolutionary relationships of *Rickettsia* spp. *gltA*. The evolutionary history was inferred using the Neighbor-Joining method [38]. The bootstrap consensus tree inferred from 10,000 replicates is taken to represent the evolutionary history of the taxa analyzed [39]. Branches corresponding to partitions reproduced in less than 50% bootstrap replicates are collapsed. The percentage of replicate trees in which the associated taxa clustered together in the bootstrap test (10,000 replicates) are shown next to the branches [39]. The evolutionary distances were computed using the Kimura 2-parameter method and are in the units of the number of base substitutions per site [40]. The analysis involved 18 nucleotide sequences. Codon positions included were 1st+2nd+3rd+Noncoding. All ambiguous positions were removed for each sequence pair. There were a total of 1254 positions in the final dataset. Evolutionary analyses were conducted in MEGA7 [41].

All individual adult ticks and larval/nymphal tick pools were PCR-positive for *Rickettsia* spp. Subsequent PCR testing of paired eggs resulted in 91% (30/33) PCR positive among tick egg pools for *Rickettsia* spp. Sequencing data revealed *Rickettsia* spp. shared ≥99% identity with *R*. *raoultii ompA* (Accession numbers MH234455 and MH234456) shown in the phylogenetic analysis (Fig 4). A majority (23/32) of *gltA* sequences shared ≥99% identity with *R*. *raoultii* (Accession numbers MH208721 and MH208722) shown in the phylogenetic analysis (Fig 5), however 9/32 sequences were considered inconclusive, falling between 84%-95% identity with *R*. *raoultii*.

**Fig 6:**
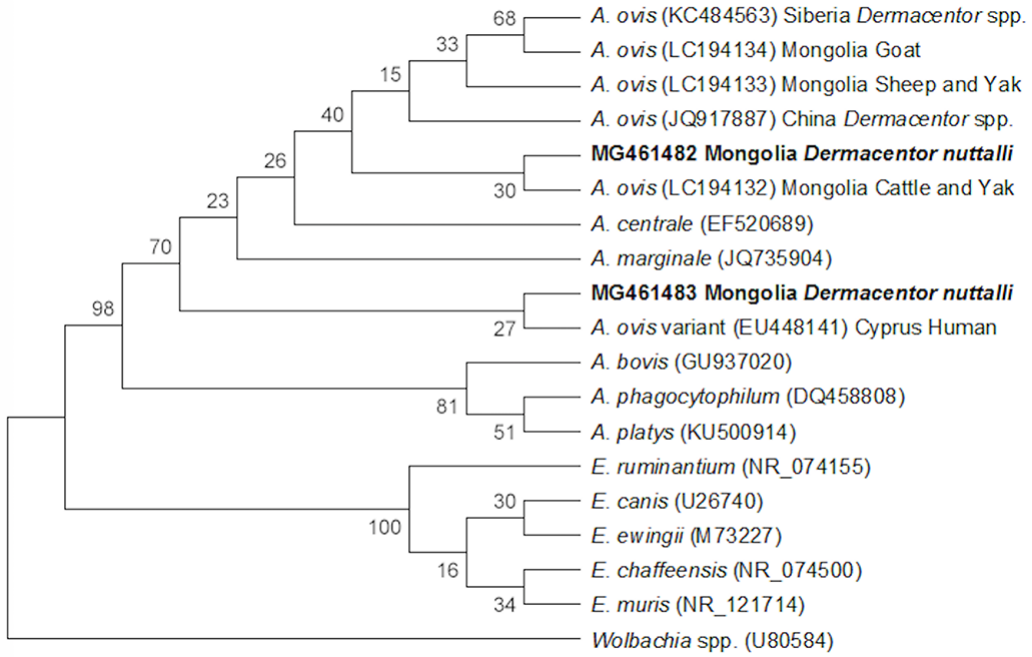
Evolutionary relationships of *Anaplasma* spp. 16S rRNA gene. The evolutionary history was inferred using the Neighbor-Joining method [38]. The bootstrap consensus tree inferred from 10,000 replicates is taken to represent the evolutionary history of the taxa analyzed [39]. Branches corresponding to partitions reproduced in less than 50% bootstrap replicates are collapsed. The percentage of replicate trees in which the associated taxa clustered together in the bootstrap test (10,000 replicates) are shown next to the branches [39]. The evolutionary distances were computed using the Kimura 2-parameter method and are in the units of the number of base substitutions per site [40]. The analysis involved 19 nucleotide sequences. Codon positions included were 1st+2nd+3rd+Noncoding. All ambiguous positions were removed for each sequence pair. There were a total of 1518 positions in the final dataset. Evolutionary analyses were conducted in MEGA7 [41].

Of the 38 engorged adult ticks collected, two ticks (5%) were PCR-positive for *Anaplasma/Ehrlichia* spp., while none of the larval/nymphal pools were PCR-positive for *Anaplasma*/*Ehrlichia* spp. Additionally, one pool of eggs laid by an *Anaplasma/Ehrlichia* spp.-positive engorged adult female tick, was also found to be PCR-positive for *Anaplasma/Ehrlichia* spp. All PCR-positive *Anaplasma/Ehrlichia* spp. ticks and the positive egg clutch were further examined using a sequencing approach to identify the infecting rickettsial species. Sequencing results indicated that the *Anaplasma/Ehrlichia* spp. positive egg clutch and corresponding engorged adult female tick shared 99% identity (accession number MG461482) and the other engorged adult female tick shared 89% (accession number MG461483) identity with the *A*. *ovis* 16S ribosomal RNA gene. Both *Anaplasma* spp. sequences are shown in the phylogenetic analysis (Fig 6).

## Discussion

Few studies have evaluated transovarial transmission of tick-borne rickettsiae in Mongolia [42, 43]. As a result, data regarding transovarial transmission of *Rickettsia* spp. [44] are particularly sparse and it remains unclear what role transovarial transmission of *Rickettsia* spp. and *Anaplasma/Ehrlichia* spp. plays in the maintenance of tick-borne rickettsiae in Mongolia. Additionally, few studies have assessed tick-borne rickettsiae in tick larvae collected from small mammals [45, 46].

It is often difficult to compare field surveys on tick-borne rickettsiae, because of varying sampling methods and sample sizes. However, the detection of *R*. *raoultii* found in this study show similarity with other studies conducted in Mongolia with 100% of *Dermacentor* spp. tick pools testing positive for *R*. *raoultii* [20, 22]. The positive molecular status of the majority of adult ticks were *R*. *raoultii* with few other ticks carrying uncharacterized rickettsiae, suggesting that the prevalence of *R*. *raoultii* in the areas in which the ticks were collected is quite high.

Though this study does not report animal host infection status, it can prove advantageous to test larvae collected from animal hosts as a method of determining if rickettsiae infect ticks by transovarial or transstadial transmission with paired animal health information. A positive infection status in ticks could occur by horizontal transmission of rickettsiae from an infected animal host to the tick. The alternative explanation is that the larvae found on the small mammals may be infected with the *Rickettsia* spp. by transovarial transmission. Transovarial transmission of *Rickettsia* spp. has been demonstrated in controlled laboratory settings and observed in nature. Laboratory demonstration of transovarial transmission has often included controlled concentration of the rickettsiae, artificial climactic conditions (temperature, humidity, etc.), and use of experimental hosts such as rabbits [44], mice, guinea pigs or capillary feeding [47]. Among these laboratory-based transovarial studies, the transmission rate of *Rickettsia* spp. from mother to progeny has been shown to occur up to 100% across various tick species, including *Dermacentor* genus [44, 48]. Additionally, many laboratory-based transovarial studies have demonstrated the efficiency of transovarial transmission of *Rickettsia* spp. over multiple generations of ticks [44, 49]. Among studies observing naturally infected ticks transmitting *Rickettsia* spp. to progeny, prevalence has ranged from 30% to 100% [48]. Though laboratory challenge studies determine the capability of transovarial transmission, observational studies of transovarial events can provide a risk assessment for transovarial transmission of tick-borne rickettsiae in a given region.

It is well documented that naturally occurring *Rickettsia* spp. are sustained through both transovarial and transstadial transmission in *Dermacentor* spp. ticks based on previous epidemiological research of *Rickettsia* spp. in Inner Mongolia, China, suggesting that there may be similar maintenance of *Rickettsia* spp. in Mongolia [50]. Previous challenge studies have demonstrated transovarial transmission of *R*. *raoultii* in *D*. *nuttalli*, *D*. *silvarum*, *D*. *marginatus*, and *D*. *reticulatus* ticks. Transovarial transmission ranged from 43% to 100% prevalence depending on generation of tick infection, species of tick, and strain of *R*. *raoultii*. In comparison to the previous laboratory-based research reporting 43% to 99.5% of transovarial transmission of *R*. *raoultii* in *D*. *nuttalli* ticks [49], this study reports comparable transovarial transmission prevalence between adult and pooled eggs in *D*. *nuttalli* ticks at 91%. Though there has been no conclusive data reporting vertebrate hosts as a reservoir for *R*. *raoultii*, it has been suggested that *D*. *marginatus* and *D*. *reticulatus* serve as both vector and reservoir [51]. Additionally, *D*. *nuttalli* ticks have been implicated as the primary vector for *R*. *raoultii* in Mongolia [20].

Our study’s high prevalence of infected egg clutches suggesting transovarial transmission in conjunction with the substantial number of infected larvae found on small mammals, suggests that *Dermacentor* species (potentially *D*. *nuttalli*) ticks may serve not only as the primary vector, but also as the primary reservoir for *R*. *raoultii* in the northern region of Mongolia.

Though this study did not identify *Anaplasma* spp. or *Ehrlichia* spp. in larvae or nymphs, there was detection of an *Anaplasma* spp. most similar to *A*. *ovis* in two *D*. *nuttalli* fed adult ticks and in one tick egg pool. There have been reports of *A*. *phagocytophilum* transmitted transovarially with prevalence’s ranging from 10% to 40% [52], however transovarial transmission of most *Anaplasma* spp. and *Ehrlichia* spp. are thought to occur at low frequencies or not at all [53, 54]. There has been research evaluating the role of transovarial transmission of *Anaplasma* spp. and *Ehrlichia* spp. with little success [55]. To our knowledge, this may be the first documented report suggesting potential transovarial transmission of *A*. *ovis* in *D*. *nuttalli* ticks [56]. There has been reports of this particular *Anaplasma* spp. in *Dermacentor* spp. ticks [57] being associated with history of infection in goats [11, 12], sheep [11, 14], cattle [11], and reindeer [16] throughout Mongolia and China. Though *A*. *ovis* is not known to cause human disease, the economic burden is great for individuals who rely on raising livestock for their income [58]. Further research is needed to evaluate the efficiency and role of potential transovarial transmission of *A*. *ovis*.

### Limitations

Like many tick pool studies, it is difficult to determine the exact prevalence of disease using this approach. Due to the nature of the maximum likelihood estimation calculation, the proportion of infected ticks with maximum likelihood of being *Rickettsia* spp. infected within tick pools cannot be calculated if 100% of sample pools are positive [59]. Additionally, due to the pooling of tick eggs, this study was unable to determine a more precise proportion of transovarial transmission from an infected female tick to at least one progeny. Though data suggest that transovarial transmission for *R*. *raoultii* did occur, we were unable to determine how many progeny were infected. Additionally, by only screening infection status of egg mass, we are unable to discuss if infected larvae will hatch. Furthermore, *Rickettsia* spp. PCR primers have been shown to cross-react with *Anaplasma* spp. and *Ehrlichia* spp. However, this study also utilized a general screening assay for *Anaplasma/Ehrlichia* spp. and confirmation by sequencing, which allowed for greater confidence in the *Rickettsia* spp. PCR assay.

### Conclusion

The indication that *D*. *nuttalli* ticks can serve as reservoirs for *R*. *raoultii* may warrants additional evaluation of transovarial and transstadial transmission of *R*. *raoultii*. Studies should focus on assessing tick eggs, either in smaller egg pools or individually, to determine the proportion of transovarial transmission as well as transstadial transmission for *R*. *raoultii* in eggs entering larval life stage, and larvae entering nymphal stages in the natural foci of Mongolia. Additionally, the testing of larvae from animal hosts and the environment should be further examined, preferably testing individual ticks instead of tick pools. Also, this report has identified a potentially novel transovarial transmission of *A*. *ovis*. Further investigation would be needed to determine the efficiency and prevalence of transovarial transmission of this rickettsiae.

## Supporting information

S1 Dataset Larvae and nymph pool data. (PDF)

S2 Dataset Engorged adult tick data. (PDF)
