# Supplementary material for: Evidence for transovarial transmission of tick-borne rickettsiae circulating in Northern Mongolia
Source: PLoS Negl Trop Dis. 2018 Aug 27;12(8):e0006696. doi: 10.1371/journal.pntd.0006696 (PMC6128658; doi:10.1371/journal.pntd.0006696)
Supplement: S1 Dataset — (PDF) [file pntd.0006696.s001.pdf]

ID	Pool Size	Genus	Species	Location		Host	
				Soum	Aimag	Scientific Name	Common Name
**L1**	1	*Dermacentor*	Unknown	Bayangol	Selenge	*Spermophilus dauricus*	Ground Squirrel
**L2**	6	*Dermacentor*	Unknown	Bayangol	Selenge	*Meriones unguiculatus*	Mongolian Gerbil
**L3**	15	*Dermacentor*	Unknown	Bayangol	Selenge	*Spermophilus dauricus*	Ground Squirrel
**L4**	15	*Dermacentor*	Unknown	Khongar	Darkahan-Uul	*Spermophilus dauricus*	Ground Squirrel
**L5**	15	*Dermacentor*	Unknown	Khongar	Darkahan-Uul	*Spermophilus dauricus*	Ground Squirrel
**L6**	15	*Dermacentor*	Unknown	Khongar	Darkahan-Uul	*Spermophilus dauricus*	Ground Squirrel
**L7**	10	*Dermacentor*	Unknown	Khongar	Darkahan-Uul	*Spermophilus dauricus*	Ground Squirrel
**L8**	10	*Dermacentor*	Unknown	Khongar	Darkahan-Uul	*Spermophilus dauricus*	Ground Squirrel
**L9**	15	*Dermacentor*	Unknown	Khongar	Darkahan-Uul	*Cricetulus barabensis*	Striped Dwarf Hamster
**L10**	5	*Dermacentor*	Unknown	Orkhon	Darkahan-Uul	*Spermophilus dauricus*	Ground Squirrel
**L11**	15	*Dermacentor*	Unknown	Eruu	Selenge	*Cricetulus barabensis*	Striped Dwarf Hamster
**L12**	15	*Dermacentor*	Unknown	Eruu	Selenge	*Cricetulus barabensis*	Striped Dwarf Hamster
**L13**	15	*Dermacentor*	Unknown	Eruu	Selenge	*Cricetulus barabensis*	Striped Dwarf Hamster
**L14**	15	*Dermacentor*	Unknown	Eruu	Selenge	*Cricetulus barabensis*	Striped Dwarf Hamster
**L15**	13	*Dermacentor*	Unknown	Eruu	Selenge	*Spermophilus dauricus*	Ground Squirrel
**L16**	15	*Dermacentor*	Unknown	Eruu	Selenge	*Cricetulus barabensis*	Striped Dwarf Hamster
**L17**	15	*Dermacentor*	Unknown	Eruu	Selenge	*Cricetulus barabensis*	Striped Dwarf Hamster
**L18**	15	*Dermacentor*	Unknown	Eruu	Selenge	*Cricetulus barabensis*	Striped Dwarf Hamster
**L19**	15	*Dermacentor*	Unknown	Eruu	Selenge	*Cricetulus barabensis*	Striped Dwarf Hamster
**L20**	15	*Dermacentor*	Unknown	Eruu	Selenge	*Cricetulus barabensis*	Striped Dwarf Hamster
**L21**	15	*Dermacentor*	Unknown	Eruu	Selenge	*Cricetulus barabensis*	Striped Dwarf Hamster
**L22**	15	*Dermacentor*	Unknown	Eruu	Selenge	*Cricetulus barabensis*	Striped Dwarf Hamster
**L23**	11	*Dermacentor*	Unknown	Yavkhlant	Selenge	*Tamius sibiricus*	Siberian Chipmunk
**L24**	10	*Dermacentor*	Unknown	Yavkhlant	Selenge	*Tamius sibiricus*	Siberian Chipmunk
**L25**	15	*Dermacentor*	Unknown	Yavkhlant	Selenge	*Spermophilus dauricus*	Ground Squirrel
**L26**	15	*Dermacentor*	Unknown	Yavkhlant	Selenge	*Spermophilus dauricus*	Ground Squirrel
**L27**	15	*Dermacentor*	Unknown	Yavkhlant	Selenge	*Spermophilus dauricus*	Ground Squirrel
**L28**	15	*Dermacentor*	Unknown	Yavkhlant	Selenge	*Spermophilus dauricus*	Ground Squirrel
**L29**	15	*Dermacentor*	Unknown	Yavkhlant	Selenge	*Spermophilus dauricus*	Ground Squirrel
**L30**	15	*Dermacentor*	Unknown	Yavkhlant	Selenge	*Spermophilus dauricus*	Ground Squirrel
**L31**	6	*Dermacentor*	Unknown	Yavkhlant	Selenge	*Spermophilus dauricus*	Ground Squirrel
**L32**	15	*Dermacentor*	Unknown	Unknown	Darkahan-Uul	*Meriones unguiculatus*	Mongolian Gerbil
**L33**	1	*Dermacentor*	Unknown	Unknown	Tuv	*Apodemus peninsulae*	Korean Field Mouse
**L34**	15	*Dermacentor*	Unknown	Batsumber	Tuv	*Cricetulus barabensis*	Striped Dwarf Hamster
**L35**	15	*Dermacentor*	Unknown	Batsumber	Tuv	*Cricetulus barabensis*	Striped Dwarf Hamster
**L36**	6	*Dermacentor*	Unknown	Batsumber	Tuv	*Spermophilus dauricus*	Ground Squirrel
**L37**	15	*Dermacentor*	Unknown	Batsumber	Tuv	*Spermophilus dauricus*	Ground Squirrel
**L38**	15	*Dermacentor*	Unknown	Batsumber	Tuv	*Cricetulus barabensis*	Striped Dwarf Hamster
**L39**	5	*Dermacentor*	Unknown	Batsumber	Tuv	*Cricetulus barabensis*	Striped Dwarf Hamster
**L40**	5	*Dermacentor*	Unknown	Batsumber	Tuv	*Meriones unguiculatus*	Mongolian Gerbil
**L41**	11	*Dermacentor*	Unknown	Batsumber	Tuv	*Ochotona*	Pika
**L42**	11	*Dermacentor*	Unknown	Batsumber	Tuv	*Ochotona*	Pika
**N1**	1	*Dermacentor*	Unknown	Khongar	Darkahan-Uul	*Spermophilus dauricus*	Ground Squirrel
**N2**	1	*Dermacentor*	Unknown	Eruu	Selenge	*Spermophilus dauricus*	Ground Squirrel
**N3**	1	*Dermacentor*	Unknown	Eruu	Selenge	*Cricetulus barabensis*	Striped Dwarf Hamster
**N4**	1	*Dermacentor*	Unknown	Yavkhlant	Selenge	*Spermophilus dauricus*	Ground Squirrel
**N5**	5	*Dermacentor*	Unknown	Batsumber	Tuv	*Spermophilus dauricus*	Ground Squirrel
**N6**	5	*Dermacentor*	Unknown	Batsumber	Tuv	*Spermophilus dauricus*	Ground Squirrel
**N7**	5	*Dermacentor*	Unknown	Batsumber	Tuv	*Spermophilus dauricus*	Ground Squirrel
**N8**	5	*Dermacentor*	Unknown	Batsumber	Tuv	*Spermophilus dauricus*	Ground Squirrel
**N9**	5	*Dermacentor*	Unknown	Batsumber	Tuv	*Spermophilus dauricus*	Ground Squirrel
**N10**	5	*Dermacentor*	Unknown	Batsumber	Tuv	*Spermophilus dauricus*	Ground Squirrel
**N11**	5	*Dermacentor*	Unknown	Batsumber	Tuv	*Spermophilus dauricus*	Ground Squirrel
**N12**	3	*Dermacentor*	Unknown	Batsumber	Tuv	*Spermophilus dauricus*	Ground Squirrel
**N13**	5	*Dermacentor*	Unknown	Batsumber	Tuv	*Ochotona*	Pika
**N14**	5	*Dermacentor*	Unknown	Batsumber	Tuv	*Ochotona*	Pika
**N15**	5	*Dermacentor*	Unknown	Batsumber	Tuv	*Ochotona*	Pika
**N16**	5	*Dermacentor*	Unknown	Batsumber	Tuv	*Ochotona*	Pika
**N17**	5	*Dermacentor*	Unknown	Batsumber	Tuv	*Ochotona*	Pika
**N18**	5	*Dermacentor*	Unknown	Batsumber	Tuv	*Ochotona*	Pika
