# Supplementary material for: Evidence for transovarial transmission of tick-borne rickettsiae circulating in Northern Mongolia
Source: PLoS Negl Trop Dis. 2018 Aug 27;12(8):e0006696. doi: 10.1371/journal.pntd.0006696 (PMC6128658; doi:10.1371/journal.pntd.0006696)
Supplement: S2 Dataset — (PDF) [file pntd.0006696.s002.pdf]

ID	Genus	Species	Sex	Location		Host	Eggs (mg)
				Soum	Aimag		
ET01	*Dermacentor*	*nuttalli*	Female	Tarialan	Khuvsgul	Sheep	100
ET02	*Dermacentor*	*nuttalli*	Female	Tarialan	Khuvsgul	Sheep	270
ET03	*Dermacentor*	*nuttalli*	Female	Tarialan	Khuvsgul	Sheep	150
ET04	*Dermacentor*	*nuttalli*	Female	Tarialan	Khuvsgul	Sheep	10
ET05	*Dermacentor*	*nuttalli*	Female	Tarialan	Khuvsgul	Sheep	20
ET06	*Dermacentor*	*nuttalli*	Female	Tarialan	Khuvsgul	Sheep	10
ET07	*Dermacentor*	*nuttalli*	Female	Tarialan	Khuvsgul	Sheep	190
ET08	*Dermacentor*	*nuttalli*	Female	Tarialan	Khuvsgul	Sheep	220
ET09	*Dermacentor*	*nuttalli*	Female	Tarialan	Khuvsgul	Sheep	140
ET10	*Dermacentor*	*nuttalli*	Female	Tarialan	Khuvsgul	Sheep	30
ET11	*Dermacentor*	*nuttalli*	Female	Tarialan	Khuvsgul	Sheep	90
ET12	*Dermacentor*	*nuttalli*	Female	Tarialan	Khuvsgul	Sheep	20
ET13	*Dermacentor*	*nuttalli*	Female	Tarialan	Khuvsgul	Sheep	30
ET14	*Dermacentor*	*nuttalli*	Female	Tarialan	Khuvsgul	Sheep	30
ET15	*Dermacentor*	*nuttalli*	Female	Tarialan	Khuvsgul	Sheep	10
ET16	*Dermacentor*	*nuttalli*	Female	Tarialan	Khuvsgul	Sheep	30
ET17	*Dermacentor*	*nuttalli*	Female	Tarialan	Khuvsgul	Sheep	0
ET18	*Dermacentor*	*nuttalli*	Female	Tarialan	Khuvsgul	Sheep	20
ET19	*Dermacentor*	*nuttalli*	Female	Tumentsogt	Sukhbaatar	Cattle	40
ET20	*Dermacentor*	*nuttalli*	Female	Tumentsogt	Sukhbaatar	Cattle	290
ET21	*Dermacentor*	*nuttalli*	Female	Tumentsogt	Sukhbaatar	Cattle	240
ET22	*Dermacentor*	*nuttalli*	Female	Tumentsogt	Sukhbaatar	Cattle	230
ET23	*Dermacentor*	*nuttalli*	Female	Tumentsogt	Sukhbaatar	Cattle	190
ET24	*Dermacentor*	*nuttalli*	Female	Tumentsogt	Sukhbaatar	Cattle	230
ET25	*Dermacentor*	*nuttalli*	Female	Tumentsogt	Sukhbaatar	Cattle	150
ET26	*Dermacentor*	*nuttalli*	Female	Tumentsogt	Sukhbaatar	Cattle	30
ET27	*Dermacentor*	*nuttalli*	Female	Altnabulag	Selenge	Sheep	290
ET28	*Dermacentor*	*nuttalli*	Female	Altnabulag	Selenge	Sheep	0
ET29	*Dermacentor*	*nuttalli*	Female	Altnabulag	Selenge	Sheep	170
ET30	*Dermacentor*	*nuttalli*	Female	Altnabulag	Selenge	Sheep	0
ET31	*Dermacentor*	*nuttalli*	Female	Altnabulag	Selenge	Sheep	410
ET32	*Dermacentor*	*nuttalli*	Female	Altnabulag	Selenge	Sheep	160
ET33	*Dermacentor*	*nuttalli*	Female	Altnabulag	Selenge	Sheep	220
ET34	*Dermacentor*	*nuttalli*	Female	Altnabulag	Selenge	Sheep	30
ET35	*Dermacentor*	*nuttalli*	Female	Altnabulag	Selenge	Sheep	0
ET36	*Dermacentor*	*nuttalli*	Female	Altnabulag	Selenge	Sheep	30
ET37	*Dermacentor*	*nuttalli*	Female	Altnabulag	Selenge	Sheep	150
ET38	*Dermacentor*	*nuttalli*	Female	Altnabulag	Selenge	Sheep	0
